# Plasma mtDNA as a possible contributor to and biomarker of inflammation in rheumatoid arthritis

**DOI:** 10.1186/s13075-024-03329-2

**Published:** 2024-05-07

**Authors:** Julia Lehmann, Stavros Giaglis, Diego Kyburz, Douglas Daoudlarian, Ulrich A. Walker

**Affiliations:** 1 Laboratory for Experimental Rheumatology, Department of Biomedicine, University of Basel, Basel, Switzerland; 2 Department of Rheumatology, University Hospital Basel, Petersgraben 4, CH 4037 Basel, Switzerland

## Abstract

**Objectives:**

Neutrophil extracellular trap formation and cell-free DNA (cfDNA) contribute to the inflammation in rheumatoid arthritis (RA), but it is unknown if mitochondrial DNA (mtDNA) or nuclear DNA (nDNA) is more abundant in the circulation. It is unclear if DNA concentration measurements may assist in clinical decision-making.

**Methods:**

This single-center prospective observational study collected plasma from consecutive RA patients and healthy blood donors. Platelets were removed, and mtDNA and nDNA copy numbers were quantified by polymerase chain reaction (PCR).

**Results:**

One hundred six RA patients and 85 healthy controls (HC) were recruited. Circulating median mtDNA copy numbers were increased 19.4-fold in the plasma of patients with RA (median 1.1 x10^8^ copies/mL) compared to HC (median 5.4 x10^6^ copies/mL, *p*<0.0001). Receiver operating characteristics (ROC) curve analysis of mtDNA copy numbers identified RA patients with high sensitivity (92.5%) and specificity (89.4%) with an area under the curve (AUC) of 0.97, *p* <0.0001 and a positive likelihood ratio of 8.7.

Demographic, serological (rheumatoid factor (RF) positivity, anti-citrullinated protein antibodies (ACPA) positivity) and treatment factors were not associated with DNA concentrations. mtDNA plasma concentrations, however, correlated significantly with disease activity score-28- erythrocyte sedimentation rate (DAS28-ESR) and increased numerically with increasing DAS28-ESR and clinical disease activity index (CDAI) activity. MtDNA copy numbers also discriminated RA in remission (DAS28 <2.6) from HC (*p*<0.0001). Also, a correlation was observed between mtDNA and the ESR (*p* = 0.006, *R*= 0.29). Similar analyses showed no significance for nDNA.

**Conclusion:**

In contrast to nDNA, mtDNA is significantly elevated in the plasma of RA patients compared with HC. Regardless of RA activity, the abundance of circulating mtDNA is a sensitive discriminator between RA patients and HC. Further validation of the diagnostic value of mtDNA testing is required.

## Key messages

What is already known about this subject?

Cell free DNA is elevated in the blood circulation of RA patients compared to healthy individuals and therefore may be involved in the pathogenesis of its inflammatory process. Whether the cell-free DNA is predominantly of mitochondrial or nuclear nature is currently unknown and remains to be established.

What does this study add?

This study demonstrates a significant increase of circulating mtDNA in RA plasma that identifies RA patients with high sensitivity, irrespective of seropositivity and disease activity. Nuclear DNA, in contrast, was not elevated in RA plasma.

How might this impact on clinical practice or further developments?

Although extracellular mtDNA in plasma is a non-specific biomarker for inflammatory processes and other autoimmune diseases, mtDNA quantification, unlike nDNA, may assist in the identification of inflammatory arthritis. The proinflammatory pathways triggered by mtDNA may also evolve as a pharmacological target.

## Introduction

Rheumatoid arthritis represents the second most prevalent autoimmune disease; it is characterized by the frequent development of autoantibodies and the induction of synovitis in peripheral joints, leading to irreversible cartilage and bone destruction when not sufficiently treated [1]. The pathogenesis of RA is not fully understood but increased amounts of cfDNA were found in the blood circulation and in the joint fluid of RA patients [2–4]. These findings and other studies, in which a correlation between disease activity and the amount of cfDNA in peripheral blood was demonstrated, raise the possibility that cfDNA may maintain local and systemic inflammation in RA [4–7]. The cfDNA measured in this process, however may be of nuclear or of mitochondrial origin [7, 8]. MtDNA induces arthritis when injected into a mouse joint, whereas nDNA does not show this effect [9]. MtDNA has an abundance of hypomethylated CpG motifs and oxidative damage due to its proximity to oxygen radicals generated by the respiratory chain. Both the CpG motifs and oxidatively damaged DNA are recognized as danger-associated molecular patterns (DAMPs) by sensors of the innate immune system [10, 11]. A possible source of extracellular DNA may be neutrophilic granulocytes which, upon a variety of stimuli, may undergo neutrophil extracellular traps (NET) formation, releasing nDNA or mtDNA into the extracellular space [12–14]. Compared to healthy individuals, a large amount of neutrophils is seen in the synovial fluid of RA patients [15]. RA patients' neutrophils are prone to form NETs, with neutrophils from both the circulation and synovial fluid releasing large amounts of DNA [12, 16–18].

The studies that quantified circulating DNA in patients with RA yielded controversial results [4, 5, 19]. Some found an increase in cfDNA and mtDNA compared to the healthy population, while others have observed a decrease [7, 19, 20]. These discrepancies are likely to result partly from differences in the analyte (serum vs. plasma). Moreover, all studies lacked platelet removal. Platelets, however, release mtDNA upon activation and coagulation, and therefore, guidelines mandate platelet removal prior to cfDNA quantification to eliminate platelets as a confounder [21].

A possible impact of this study is to quantify and compare nDNA and mtDNA in the plasma of patients with RA and HC, with the intention to better understand the role of circulating nucleic acids in the pathogenesis of RA and to investigate a possible utility in the diagnosis and monitoring of RA activity.

## Methods

### Study subjects

After ethics committee approval by the regional ethical committee of Northwest and Central Switzerland EKNZ, adult patients fulfilling the American College of Rheumatology classification criteria 2010 for RA [22] were recruited at the University Hospital Basel from August 2019 until August 2021. Informed, written consent was obtained from all patients in the study before blood was taken.

Concomitant non-RA autoimmune diseases, active systemic infection, trauma, as well as untreated neoplasia were considered as known causes of extracellular DNA elevation themselves and were therefore excluded [23–26]. RA activity at the time of blood collection was assessed by DAS28-ESR, and CDAI scoring by the treating physician [27–29]. Blood pressure was measured with a systolic blood pressure above 140mmHg defined as arterial hypertension. Healthy adult volunteer blood donors serving as control groups were consecutively recruited at the Basel University blood bank or from the hospital staff. Blood donors had to fulfil the Swiss eligibility criteria for blood donation, according to which persons with systemic autoimmune diseases, current medication with immunosuppressive agents, malignant neoplasia and recovery after a major illness or surgery are excluded. All procedures were in accordance with the Helsinki Declaration of Good Clinical Practice [30].

### Isolation of total DNA from plasma and quantification of circulation DNA copy numbers

In all subjects, 4.5 mL of blood were collected in an EDTA tube by peripheral venipuncture. Within 4 hours, plasma was processed in accordance with guidelines [21]. At room temperature, blood samples were initially centrifuged at 1200xg for 10 minutes. In order to eliminate platelets (which contain mtDNA), the plasma was then transferred into another microcentrifuge tube, centrifuged again at 12000*g for 10 minutes [31], and platelet-poor plasma aspirated. Total DNA was extracted from 500 µL of platelet-poor plasma utilizing the QIAamp DNA Blood Mini kit (Qiagen, Hilden, Germany). The obtained total cfDNA was quantified by spectrophotometry NanoDrop ND-1000 (Nano‐Drop Technologies, Wilmington, DE, USA). MtDNA and nDNA copy numbers were quantified with SYBR Green on an Applied Biosystems StepOne Plus Real-Time PCR system (Thermo Scientific, Wilmington, DE, USA). The mtDNA ATP-6 gene amplification was achieved using the forward primer 5′-ACCAATAGCCCTGGCCGTAC-3′ and the reverse primer 5′-GGTGGCGCTTCCAATTAGGT-3. To detect nDNA, exon 8 of the glyceraldehyde 3-phosphate dehydrogenase (GAPDH) gene, we utilized the forward primer 5′-CGGGGCTCTCCAGAACATC-3′ and the reverse primer 5′-ATGACCTTGCCCACAGCCT-3′. A comprehensive description of the PCR procedure and its analysis was described previously [32].

### Statistical analysis

The statistical analysis was carried out with GraphPad Prism software (Version 8.4.3, GraphPad Software, Inc., La Jolla, CA, USA). Data were expressed as the median and interquartile range (IQR). Mann- Whitney U tests or Kruskal-Wallis tests were performed to evaluate statistically significant differences between groups, as appropriate. To analyze correlations, Spearman’s rank tests were applied. Within groups, variables were tested univariably and multivariably by linear regression analysis; a *P* value of <0.05 was considered statistically significant. No correction was performed for multiple statistical testing.

## Results

### Study subjects

One hundred and six RA patients were recruited. Fifty-one patients were female. The median age was 62 years (range 48-83 years), and the median disease duration was 7.7 years (range 0-53 years). Further details on the demographic, disease and treatment characteristics of the RA patients and HC are provided in Table 1.

**Table 1:** Demographics and disease characteristics of the study population

**Parameter**	**RA** **(*n*= 106)**	**HC** **(*n*=85)**
Age, median years (IQR)	62.0 (17.3)	51.0 (16)
Female, %	48.1	62.3
RA duration, median years (IQR)	7.7 (11.2)	na
Erosions present, %	50.9	na
DAS28-ESR, median (IQR)	2.5 (1.5)	na
- Remission %	54.4	
- Low disease activity %	16.7	
- Moderate disease activity %	24.4	
- High disease activity %	4.4	
CDAI, median (IQR)	6.0 (9.0)	na
- Remission %	29.1	
- Low disease activity %	35.9	
- Moderate disease activity %	25.2	
- High disease activity %	9.7	
Systolic blood pressure > 140 mmHg, %	33.7	nd
Diabetes mellitus, %	17.0	nd
Body mass index, median (IQR)	26.8 (6.8)	nd
Smokers, %	18.8	nd
**Laboratory parameters**		
ACPA positive, %	45.7	na
RF positive, %	51.9	na
Neutrophil count, median 10^9 cells/L (IQR)	4.3 (2.3)	nd
Platelet count, median 10^9 cells/L (IQR)	245.0 (112.0)	nd
CRP, median mg/L (IQR)	1.1 (4.7)	nd
ESR, median mm/h (IQR)	10.0 (16.0)	nd
Serum creatinine, median µmol/L (IQR)	75.0 (22.0)	nd
**RA treatment**		
No DMARD, %	14.2	na
1 DMARD, %	47.2	na
2 DMARDs, %	36.8	na
≥ 3 DMARDs, %	1.9	na
csDMARD, %	60.4	na
tsDMARD, %	18.9	na
bDMARD, %	43.4	na
Patients on prednisone, %	28	na
Prednisone dose, median mg (IQR)	5 (5)	na

### Circulating DNA concentrations in RA patients and HC subjects

**Fig. 1:**
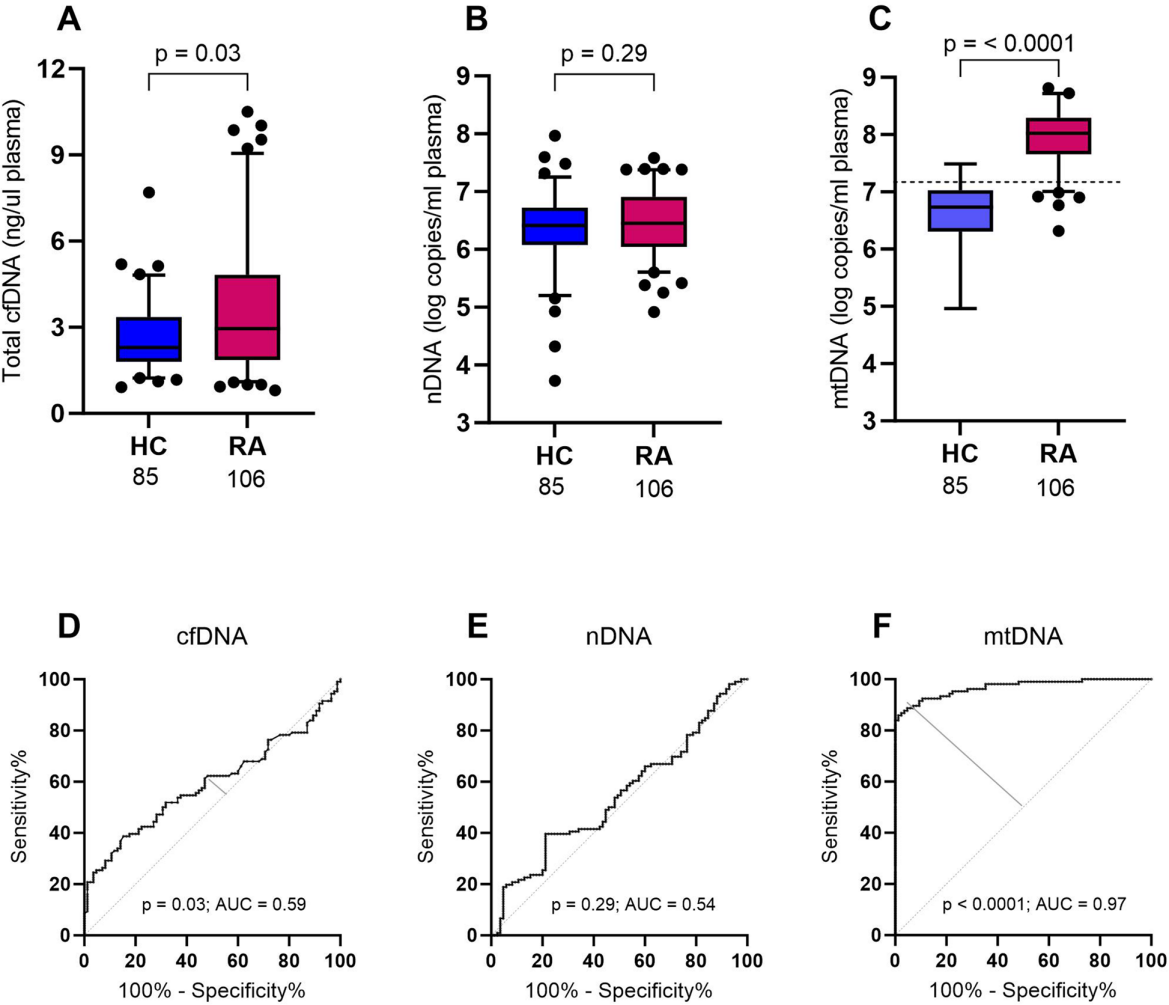
Total cfDNA (**A**) and mtDNA (**C**) in RA patients (*n*=106) are increased in comparison with HC individuals (*n*=85). nDNA (**B**) plasma levels however are not elevated in RA patients. Boxes represent IQR, whiskers represent the 5th and 95th percentile and dots represent outliers. The dotted horizontal line in panel **C** represents the optimal cut off for mtDNA testing (1.57x10^7^ copies/ml). ROC curves for total cfDNA (**D**), nDNA (**E**) and mtDNA (**F**) plasma concentrations to discriminate between HC (*n*=85) and RA patients (*n*=106). cfDNA, cell-free DNA; HC, healthy controls; mtDNA, mitochondrial DNA; nDNA, nuclear DNA; RA, rheumatoid arthritis; AUC, area under the curve

In the HC group, the median total cfDNA measured by spectrophotometry was 2.3 ng/µl (IQR: 1.6), ranging from 0.9 ng/µl to 7.7 ng/µl. In the RA patients, the median total cfDNA was 3.0 ng/µl (IQR: 3.0), ranging from 0.8 ng/µl to 10.5 ng/µl, which was significantly higher compared to HC (*p* = 0.03, Fig. 1A).

To investigate the nature of the increased cfDNA, we quantified the circulating nDNA and mtDNA copy numbers by PCR. In HC the median nDNA copy number was 2.6x10^6^ /mL plasma (IQR: 4.1 x10^6^). In RA patients, the median nDNA copy number was similar (2.8 x10^6^ copies, IQR: 7.0 x10^6^, Fig. 1B). With respect to mtDNA, the median numbers were 1.1 x10^8^ copies/mL (IQR 1.5 x10^8^) in the RA patients and 5.4 x10^6^ copies/mL (IQR 8.6 x10^6^) in the HC group. Thus, the median mtDNA copy numbers were 19.4 times higher in RA patients than in HC subjects (*p* = < 0.0001, Fig. 1C).

The potential of circulating DNA to discriminate RA subjects from HC individuals was explored in our study population of 191 subjects by means of ROC curve analysis (Fig. 1D-F). For total cfDNA, the discriminative power at the optimal cut-off of 2.32 ng/mL was relatively low (sensitivity 62.3%, specificity 51.8%), as reflected by an AUC of 0.59. Unlike nDNA copy numbers which lacked a significant difference between RA and HC, mtDNA copy numbers at a cut-off of 1.57 x 10^7,^ detected RA with high sensitivity (92.5%) and specificity (89.4%), resulting in an AUC of 0.97 and a positive likelihood ratio for a positive mtDNA test of 8.7.

### Analysis of circulating DNA by demographic and RA characteristics

In both the RA and HC groups, total cfDNA, nDNA, and mtDNA plasma concentrations did not differ between sexes (data not shown). There were no correlations between nDNA and mtDNA amounts in both the HC and RA patient groups. There were also no correlations between RA duration and cfDNA, nDNA or mtDNA levels.

CfDNA, nDNA, and mtDNA copy numbers did not differ between ACPA- positive and ACPA-negative RA patients and not between RF positive and seronegative patients. Both ACPA-negative and rheumatoid factor-negative RA patients had significantly higher mtDNA plasma concentrations than HC (*p* < 0.0001 for both comparisons).

Moreover, DNA concentrations were not correlated with neutrophil or platelet counts. A weak correlation was found between mtDNA and the ESR (*p* = 0.006, *R*= 0.29) but not with the CRP. We also found no significant difference in mtDNA plasma levels between RA patients with elevated CRP and ESR compared to those with normal inflammatory markers.

We also analyzed possible associations of DNA concentrations with cardiovascular risk factors based on the association between RA and an increased cardiovascular risk and the known correlation between cfDNA and carotid intima-media thickness [33, 34]. There were no correlations between total cfDNA, nDNA or mtDNA concentrations and body mass index or arterial blood pressure and no associations with concomitant diabetes mellitus, current smoking status and systolic arterial hypertension. There were also no associations between circulating DNA amounts and the number of cardiovascular risk factors (data not shown).

### Analysis of circulating DNA with different therapeutic modalities and prednisone

There were no significant differences in the plasma concentrations of total cfDNA, nDNA and mtDNA between RA patients who were receiving any disease-modifying antirheumatic drug (DMARD) at the time of blood collection and those who were not receiving DMARDs. There were also no significant differences in cfDNA, nDNA, and mtDNA concentrations with respect to the number and nature of the implemented DMARD therapy. There were also no correlations between the daily dose of prednisone and all DNA concentrations.

### Association and correlation of circulating DNA with RA activity

**Fig. 2:**
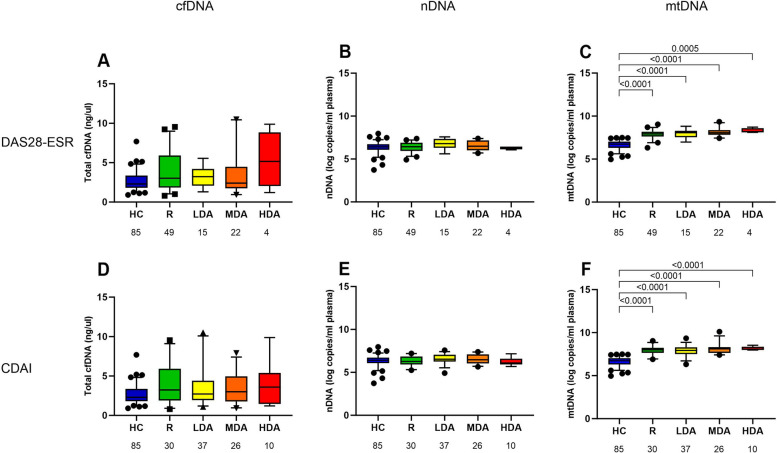
Circulating mtDNA copy numbers (**C**, **F**) but not total cfDNA levels (**A**, **D**) and nDNA copy numbers (**B**, **E**) are significantly elevated in RA patients compared with HC individuals throughout all strata of DAS28-ESR and CDAI activity. Furthermore, there is a trend of mtDNA to increase continuously with higher RA activity. Boxes represent IQR, whiskers represent the 5th and 95th percentile, and dots represent outliers. cfDNA, cell-free DNA; mtDNA, mitochondrial DNA; nDNA, nuclear DNA, DAS28, disease activity score-28; CDAI, clinical disease activity index; ESR, erythrocyte sedimentation rate; HC, healthy controls; R, remission; LDA, low disease activity; MDA, moderate disease activity; HDA, high disease activity

We finally analyzed the circulating DNA concentrations in different strata of RA activity (Fig. 2). Even in patients with the highest RA activity, cfDNA concentration as measured by spectrophotometry and nDNA copy numbers were similar to those of HC. However, mtDNA copy numbers, were significantly increased in all DAS28-ESR and CDAI strata compared to HC, and there was also a trend for an increase of mtDNA concentrations in higher RA activity strata. Interestingly, even RA patients in remission had elevated mtDNA levels compared with HC.

There was also no significant correlation of RA activity (DAS28-ESR or CDAI) with cfDNA and nDNA plasma concentrations. A significant correlation was, however, found between mtDNA copies/mL and DAS28-ESR (*p* = 0.039, *r* = 0.218). There was a numerical increase of mtDNA levels with CDAI activity as RA patients with moderate and high CDAI activity had a 3.1-fold higher mean mtDNA plasma concentration than patients in CDAI remission, but multivariable logistic regression analysis failed to demonstrate a statistical association between RA activity measures and plasma DNA concentrations in various models adjusting for disease and treatment characteristics.

## Discussion

We demonstrated an increased abundancy of cfDNA in the plasma of RA patients compared to HC individuals and showed that this increase is largely accounted for by mtDNA rather than nDNA. Previous studies found increases of nDNA in the plasma of RA patients [4, 5] and decreases of nDNA in the serum [19].

Since platelets release mtDNA in vitro during clot formation and activation [35], thereby artefactually increasing mtDNA measurements [35, 36], our analysis was unlike that of all previous investigators [4, 5, 19] done with platelet-poor plasma and not with serum thus adhering to current guidelines [21, 37]. The lack of utilization of platelet-poor plasma in the methodology of other studies may also be the reason for the discrepant results in the quantification of extracellular DNA in previous research [19]. It is likely that mtDNA is released into the extracellular space by NETosis [38–40] , and it is known that peripheral blood neutrophils of RA patients and neutrophils present in the synovial fluid have an increased capacity of NET formation [12, 16–18]. The method used in our study to obtain platelet-poor plasma removed platelets, mitochondria and microaggregates, but not microparticles and exosomes, making it impossible to exclude other sources as a contributor to the measured mtDNA [41]. In systemic lupus erythematosus (SLE), impaired degradation of extracellular DNA by deoxyribonuclease (DNase) 1 was shown to contribute to the upregulation of mtDNA in the plasma of some patients [42, 43]. The one study investigating the activity of DNase1 in RA, however, did not find an impairment [44].

In this study, mtDNA had a strong discriminative power to distinguish between RA and HC. Although our RA population is characterized by a relatively high rate of seronegative and ACPA-negative patients, perhaps reflecting a more difficult-to-diagnose arthritis population in our tertiary care setting, the ability of mtDNA measurements to distinguish between HC and RA was observed regardless of the serological status of the RA patients. Thus, mtDNA might evolve as a sensitive biomarker in the diagnosis of RA, especially in patients with absent RF and ACPA. However, plasma mtDNA is a non-specific marker, as it is also markedly elevated in other autoimmune diseases, infections, neoplasia and trauma [23–26, 32, 45].

Interestingly, mtDNA correlated with RA activity (DAS28-ESR) although there was only a trend for higher mtDNA values in higher DAS28-ESR and CDAI activity strata. We cannot exclude the possibility that the latter lack of significance results from the high number of patients in remission and the small number of patients with high activity in our study. Other investigators have suggested a significant correlation between elevated plasma cfDNA levels and DAS28 [6, 44]. It must be determined in further studies, if mtDNA quantification has a potential in the monitoring of longitudinal changes in RA activity, as demonstrated for SLE [32] and anti-citrullinated protein antibodies (ANCA)-associated vasculitis [45].

Our findings suggest that circulating mtDNA could contribute to inflammation and, therefore, may be directly involved in the pathogenesis of RA. The proinflammatory properties of mtDNA result in part from its increased content of unmethylated CpG motifs, which are recognized as a danger signal by Toll-like receptor (TLR)-9 [10, 46]. Hydroxychloroquine, a known inhibitor of TLR-9, is an effective DMARD in the treatment of RA, supporting the pathogenetic involvement of DNA sensors in the maintenance of inflammation in RA [47]. The proinflammatory properties of mtDNA may also depend on its oxidative damage [48], which stimulates the cyclic GMP-AMP synthase- Stimulator of interferon genes (cGAS-STING) pathway to enhance the secretion of tumor necrosis factor alpha (TNF-α) and interleukin-6 (IL-6), cytokines that are targets in the treatment of RA [10, 20, 49]. Finally, mtDNA has been shown to increase neutrophil Receptor Activator of NF-κB Ligand (RANKL) expression in synovial fluid, possibly contributing to the typical erosive joint damage in RA, as RANKL is the key cytokine that activates osteoclasts, which in turn lead to bone destruction [20, 50].

## Conclusions

In summary, our study demonstrates an increased abundancy of cfDNA in the plasma of RA patients compared to HC individuals. This increase of cfDNA is accounted for by a significant increase in mtDNA. MtDNA quantification enables to discriminate RA patients from HC with a high sensitivity and specificity regardless of the RA seropositivity and disease activity. Nuclear DNA, in contrast, was not elevated in RA plasma and did not show any discriminating power. MtDNA quantification in the plasma may assist in the early diagnosis of RA, especially in patients with absent RF and ACPA and its proinflammatory pathways may evolve as a pharmacological target.

## Data Availability

No datasets were generated or analysed during the current study.

## Abbreviations

ACPA: Anti-citrullinated protein antibodies
ANCA: Antineutrophil cytoplasmic antibody
AUC: Area under the curve
CDAI: Clinical disease activity index
CfDNA: Cell-free DNA
cGAS-STING: Cyclic GMP-AMP synthase- Stimulator of interferon genes
DAS28-ESR: Disease activity score28- Erythrocyte sedimentation rate
DAMPs: Danger-associated molecular patterns
DMARD: Disease-modifying antirheumatic drug
DNase: Deoxyribonuclease
GAPDH: Glyceraldehyde 3-phosphate dehydrogenase
HC: Healthy controls
HDA: High disease activity
IL-6: Interleukin-6
IQR: Interquartile range
LDA: Low disease activity
MDA: Moderate disease activity
MtDNA: Mitochondrial DNA
NDNA: Nuclear DNA
NET: Neutrophil extracellular traps
PCR: Polymerase chain reaction
R: Remission
RA: Rheumatoid arthritis
RF: Rheumatoid factor
RANKL: Receptor Activator of NF-κB Ligand
ROC: Receiver operating characteristic
SLE: Systemic lupus erythematosus
TNF-α: Tumor necrosis factor alpha
TLR: Toll-like receptor
